# Tissue-specific methylation profile in obese patients with type 2 diabetes before and after Roux-en-Y gastric bypass

**DOI:** 10.1186/s13098-017-0214-4

**Published:** 2017-02-22

**Authors:** Priscila Sala, Raquel Susana Matos de Miranda Torrinhas, Danielle Cristina Fonseca, Graziela Rosa Ravacci, Dan Linetzky Waitzberg, Daniel Giannella-Neto

**Affiliations:** 1 FMUSP—Department of Gastroenterology, Digestive Surgery Discipline, School of Medicine, University of São Paulo (LIM 35), São Paulo, Brazil; 2 University Nove de Julho, São Paulo, Brazil

**Keywords:** Epigenetic, DNA methylation, Type 2 diabetes, Obesity, Bariatric surgery

## Abstract

Eating habits, lifestyles, and exposure to specific environmental factors can greatly impact the risk of developing type 2 diabetes (T2D), influence the genome epigenetically, and affect the expression of genes, including genes related to glycemic control, at any stage of life. The epigenetic mechanism underlying obesity and T2D pathogenesis remains poorly understood. Conventional strategies for the treatment of obesity and its comorbidities often have poor long-term adherence, and pharmacological interventions are limited. Bariatric surgery is the most effective current option to treat severe obesity, and Roux-en-Y gastric bypass (RYGB) is the most applied technique worldwide. Epigenetic changes differ depending on the approach used to treat obesity and its associated comorbidities (clinical or surgical). Compared to primary clinical care, bariatric surgery leads to much greater loss of body weight and higher remission rates of T2D and metabolic syndrome, with methylation profiles in promoter regions of genes in obese individuals becoming similar to those of normal-weight individuals. Bariatric surgery can influence DNA methylation in parallel with changes in gene expression pattern. Changes in clinical biomarkers that reflect improvements in glucose and lipid metabolism after RYGB often occur before major weight loss and are coordinated by surgery-induced changes in intestinal hormones. Therefore, the intestine methylation profile would assist in understanding the mechanisms involved in improved glycemic control after bariatric surgery. The main objectives in this area for the future are to identify epigenetic marks that could be used as early indicators of metabolic risk, and to develop treatments able to delay or even reverse these epigenetic changes. Studies that provide the “human epigenetic profile” will be of considerable value to identify tissue-specific epigenetic signatures and their role in the development of chronic diseases. Further studies should apply methods based on global analysis of the genome to identify methylated sites associated with disease and epigenetic marks associated with the remodeling response to bariatric surgery. This review describes the main epigenetic alterations associated with obesity and T2D and the potential role of RYGB in remodeling these changes.

## Background

Epigenetics concerns mechanisms for modifying gene expression at the transcriptional level, by chemical modifications of DNA and RNA chromatin. These modifications are heritable by meiotic or mitotic processes, impact gene function, and cannot be explained by changes in the DNA sequence [1]. Environmental factors may alter phenotypes by modifying gene expression, but not gene sequence, leading to epigenetic modifications [1]. Epigenetics changes include alterations in DNA methylation, chromatin, and posttranslational modifications of histones and micro RNAs. They can deregulate up to 6–10% of the genes in some cells and are associated with disease pathophysiology [2, 3]. The importance of epigenetic processes in human disease was first identified in the pathogenesis of cancer in 1980 [2]. More recently, there have been reports of the relevant roles of DNA methylation in obesity and type 2 diabetes (T2D) development [2].

In contrast to gene changes, epigenetic modifications are reversible. This characteristic may allow for the identification of novel therapeutics based on functional gene restoration. Therefore, an understanding of epigenetic mechanisms can be clinically relevant for the treatment of some chronic diseases [2, 4]. This review describes the compilation of observations from experimental (donated organs) and clinical studies published in the databases PUBMED, SCIELO, MEDLINE, SCOPUS, WEB OF SCIENCE and LILACS that focused on epigenetic alterations associated with obesity and T2D and the potential role of Roux-en-Y gastric bypass (RYGB) in remodeling these changes, as an effective therapeutic tool for the treatment of severe obesity and its associated comorbidities.

## Main text

### DNA methylation

All of the cells in a given organism’s tissues have identical DNA. This genetic material contains key information to perform any function, although not all genes are expressed at once. Moreover, DNA does not determine all of the cells’ features. Gene regulation, through epigenetic marks in the DNA or cell nucleus proteins, allow each cell to acquire its specific function [5]. Environmental factors can affect individual phenotypes by inducing epigenetic changes, which alter the DNA chromatin structure and gene accessibility to transcriptional machinery [6].

**Fig. 1:**
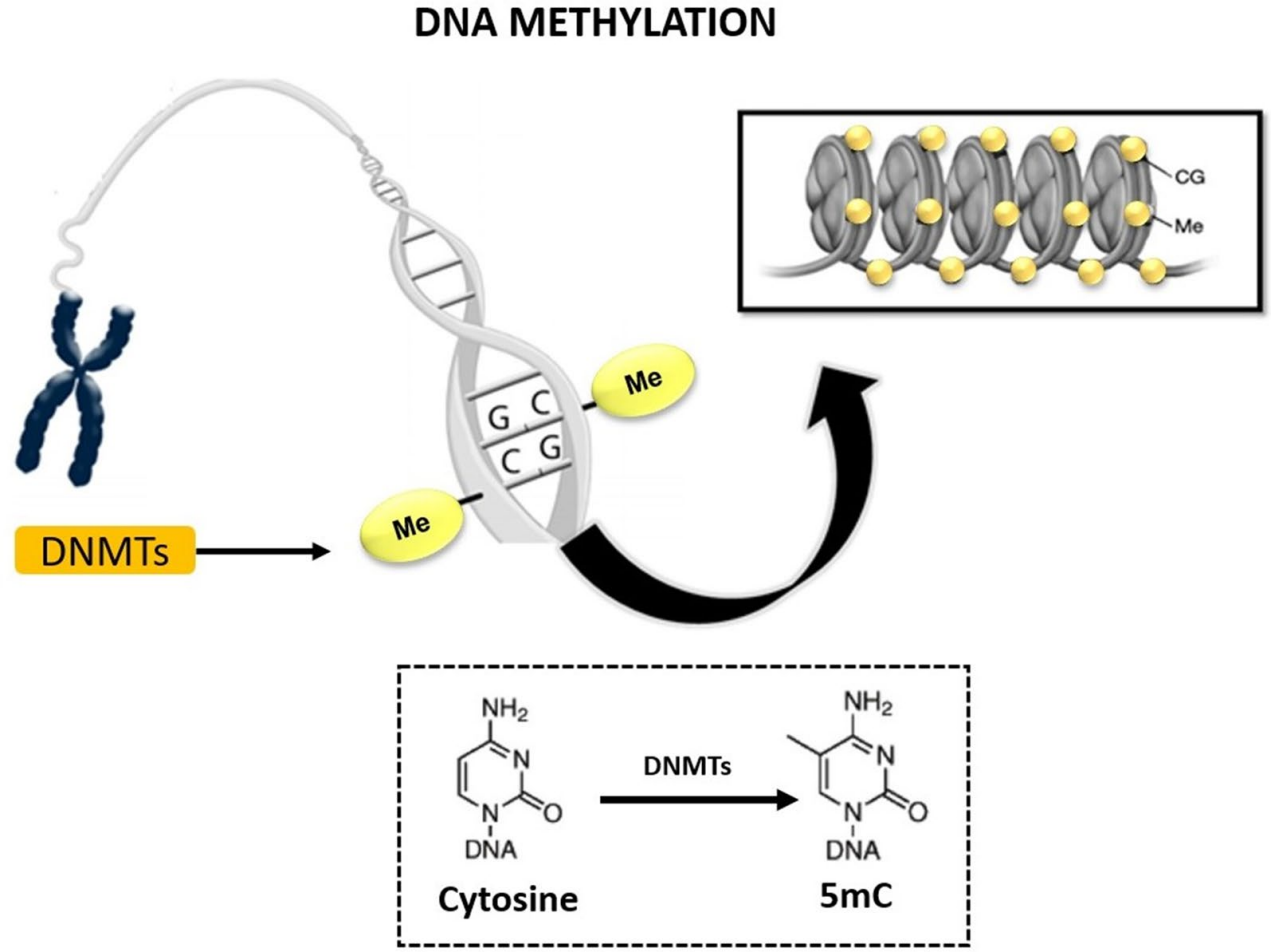
DNA methylation of mammalian genomes

One of the many epigenetic events to influence gene expression, DNA methylation consists of the covalent addition of a methyl radical (CH_3_) to a cytosine base in DNA, converting cytosine into 5-methylcytosine (5mC, Fig. 1) [7]. This reaction is catalyzed by DNA methyltransferases (DNMTs), such as DNMT1, DNMT3A, and DNMT3B [8]. DNMT1 is involved in the maintenance of DNA methylation patterns during cell division. DNMT3A and DNMT3B are de novo methyltransferases that are highly expressed in embryonic stem cells due to the elevated rate of de novo methylation at this stage [8–11].

Methylation of mammalian genomes occurs almost exclusively at cytosine linked to guanine residues through phosphodiester bonds at 5′-cytosine-phosphate-guanine (CpG) sequences, termed dinucleotides or CpG islands. There are approximately 29,000 CpG islands in the human genome [6]. CpG islands surround the promoters of constitutively expressed genes, which are demethylated [12]. CpG methylation can partially or completely prevent expression of the adjacent gene. Therefore, the transcription rate of genes containing the 5′-CpG region is often inversely proportional to their methylation [12].

The DNA methylation pattern is not uniform among eukaryotes or between species [13]. Methylation maps in human cell lines reveal that approximately 5% of all cytosine residues are methylated under normal physiological conditions [12]. As the primary function of DNA methylation is to silence active genes in DNA regions, high levels of DNA methylation are found in centromeres, telomeres, and inactive X chromosomes [14]. However, DNA methylation can occur in various locations within a gene, including the promoter region, exons, introns, and non-translated regions [15]. Non-CpG methylation also has been observed in humans [16], predominantly in embryonic stem cells [13].

The influence of DNA methylation on gene expression appears to depend on where methylation takes place in the gene sequence [17]. Methylation in promoter regions prevents transcription; methyl groups must be removed for reactivation of gene transcription [5]. Although not fully understood, gene methylation in intragenic regions appears to increase rather than silence gene activity. Actively transcribed genes exhibit high methylation levels in intragenic regions that favor mutations and are often associated with cancer [18].

**Fig. 2:**
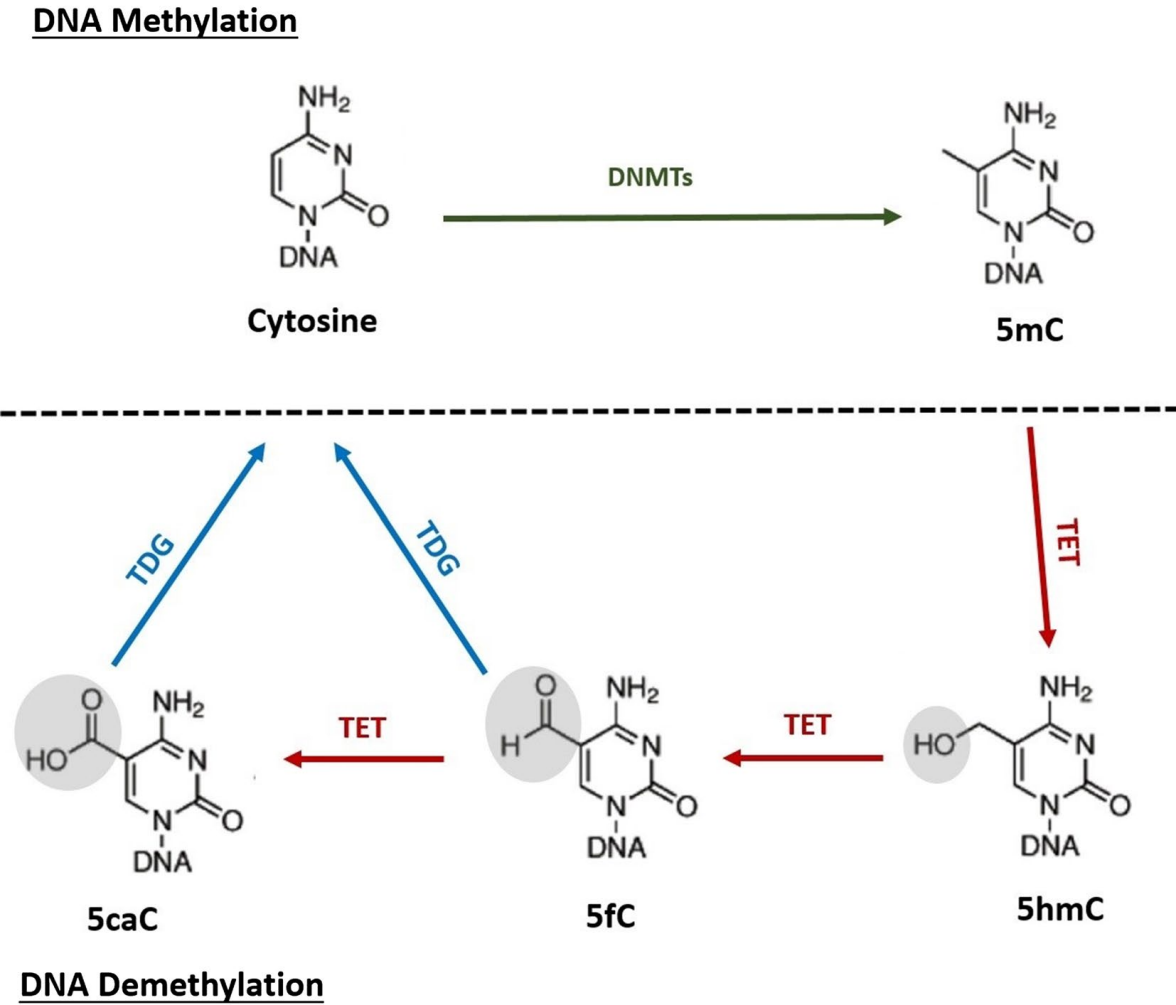
Mammalian DNA demethylation process (modified from [19]). Cytosine may be methylated by DNMTs and affect gene transcription. In the process of demethylation, the methylated product 5mC can be actively reversed by TET proteins and TDG, thereby reverting to the cytosine form. Intermediate products include 5hmC, 5FC, and 5caC. Amount of 5hmC reflects DNA demethylation status

A key discovery in epigenetics science was that DNA 5mC may be oxidized by a family of α-ketoglutarate–dependent oxygenases known as ten-eleven translocation (TET) proteins. These proteins originate 5-hydroxymethylcytosine (5hmC), 5-formylcytosine (5FC), and 5-carboxylcytosine (5caC). Thymine DNA glycosylase (TDG) can further process these 5mC derivatives, which are submitted to base excision repair or replication-dependent dilution, thereby leading to DNA demethylation [9]. The 5mC oxidation products are later reversed by unknown decarboxylases or removed by TDG and replaced with intact cytosines (Fig. 2) [10, 11]. Exact mechanisms by which methyl groups are removed and other factors remain unclear.

In short, epigenetic marks, which can be reversible, may change the way that gene transcription is controlled in the cell. Recent discoveries have increased interest in the role of epigenetic changes in the development of chronic diseases, as well as in potential therapeutics that can reverse these changes [2].

### Genetic, environmental factors, and epigenetics

Different forms of genetic variability, particularly single nucleotide polymorphisms (SNPs), have been associated with the current obesity and T2D epidemic [6]. Some polymorphisms alter protein coding in a way that impacts a range of T2D-associated pathways (i.e., synthesis, processing, and secretion of insulin; deposition of amyloid protein in pancreatic β cells; insulin resistance [IR]; and impaired gluconeogenesis regulation) but contributes little to the risk of disease development [6, 20, 21]. Human epigenotypes can be passed on through generations and predispose individuals to obesity and T2D with much greater power than any single polymorphism so far identified [6]. Eating habits, lifestyles, and exposure to specific environmental factors are commonly inherited from family. These factors can greatly impact the risk of developing T2D, influence the genome epigenetically, and affect the expression of genes, including genes related to glycemic control, at any stage of life [22].

DNA methylation varies among healthy individuals due to a combination of genetic and environmental influences and stochastic events, such as surgery for weight loss [2]. Studies investigating the stability and interindividual variations of DNA methylation in peripheral blood have compared changes in methylation profiles for relatively short (days) and long (years) periods. Some methylation marks varied significantly over time, representing markers influenced by the environment. Other epigenetic marks were highly stable, representing values determined by genetics [2, 23]. The stability and interindividual variation of DNA methylation were dependent on genomic location. Differentially methylated regions were rich in SNPs, suggesting a mechanism by which some SNPs may affect gene function [3, 23, 24].

### Epigenetics of obesity

Several studies have assessed the relation between site-specific DNA methylation and obesity. These studies focused on potential gene targets for obesity, appetite control, and insulin signaling, some of which were selected based on previous findings of altered gene expression in the same subject. Most of these studies had a cross-sectional design (e.g., DNA methylation was measured at the same time point), preventing researchers from being able to establish whether DNA methylation is a cause or a consequence of the obesity phenotype [2].

Low levels of methylation in tumor necrosis factor (*TNF*) and leptin (*LEP*) genes and high levels of methylation in proopiomelanocortin (*POMC*) and aryl hydrocarbon receptor nuclear translocator-1 (*BMAL1*) genes were identified in whole blood samples or peripheral blood leukocytes of obese subjects [25–28]. Studies demonstrated associations of body mass index (BMI) or adiposity with methylation of several genes, including pyruvate dehydrogenase kinase 4 (*PDK4*) in skeletal muscle and in type 1 receptor for melanin-concentrating hormone (*MCHR1*), serotonin transporter (*SLC6A4*), androgen receptor (*AR*), 11-beta-hydroxysteroid dehydrogenase type 2 (*HSD2*) of the period circadian clock 2 (*PER2*), and glucocorticoid receptor (*GR*) in peripheral blood leukocytes [28–33]. Taken together, these findings support the notion that obesity may be associated with epigenetic regulation of genes with a central role in intermediary metabolism.

The field of epigenetics is relatively new, and its progress has been hampered by difficulties in acquiring large numbers of tissues and homogeneous targets [34]. The recent development of next-generation technologies has allowed the global assessment of large numbers of genes and CpG islands, providing more robust data than the usual analysis targeting specific genes [2]. Technological advances have enabled the development of large-scale studies of the epigenome and its integration with genotype, transcriptome, and environment, known as epigenome-wide association studies (EWAS).

In a systematic review of 46 studies applying a global approach, the authors highlighted a lack of consistent evidence to establish a relationship between global DNA methylation and obesity [2]. However, the examined studies identified multiple methylated loci associated with obesity, especially in blood cells [25–28, 30–32, 35, 36].

Weight loss has been associated with a few changes in site-specific DNA methylation, mainly in genes involved in weight control, insulin secretion, inflammation, and circadian rhythm. Several associations have been made between methylation marks at birth and weight in later life [2]. The apparent modification of the methylation profile by weight loss suggests that some methylation marks can be a consequence of the obesity phenotype rather than programmed marks that predispose an individual to the disease [2]. Comparing the methylation profiles of obese individuals with and without success in achieving intentional weight loss after dietetic and surgical interventions would identify biomarker predictors of individual response to weight loss interventions. Potential epigenetic markers for obesity have been identified, and several of these markers are modifiable by changing the exposure in utero or the lifestyle in adult life. These findings may allow the development of targeted prevention strategies or interventions in postnatal life to modify unfavorable epigenomic profiles [2].

Recently, van Dijk et al. reviewed results obtained from larger EWAS and other studies. They found that BMI was associated with 37 methylation loci, including regions of carnitine palmitoyltransferase 1A *(CPT1A)*, ATP binding cassette subfamily G member 1 (*ABCG1*), and sterol regulatory element binding transcription factor 1 (*SREBF1*), in blood samples and with hypoxia inducible fator 3 alpha subunit (*HIF3A*) gene methylation in whole blood and adipose tissue. DNA methylation in lymphocyte antigen 86 *(LY86)* in blood leukocytes differed between lean and obese subjects. Methylation in the promoter region of proliferator-activated receptor G coactivator 1A (*PGC1A*) in the whole blood of children was associated with adiposity 5 years later. Methylation of adrenoceptor beta 3 *(ADRB3)* in whole blood was associated with waist–hip ratio. BMI and anthropometric measurements were associated with methylation in several DNA regions of adipose tissue. The authors suggested that methylation changes in *PGC1A*, *HIF3A*, *ABCG1*, and *CPT1A* emerge as biomarkers associated with metabolic health and disease [37]. In fact, EWAS have demonstrated associations between methylation of DNA regions and plasma concentrations of lipids, serum concentrations of metabolites, IR, and T2D [37].

### Epigenetics of T2D

Preventive and therapeutic management of T2D involves genetic screening [34]. Individuals who have siblings with T2D present 2–3 times higher risk of developing the disease compared to the general population [38]. For subjects who have one or both parents with T2D, the risk of developing the disease increases 30–40 or 70%, respectively [39]. A genome-wide association study (GWAS) identified at least 75 independent loci for T2D [34, 40]. However, genetic loci explain only a small proportion of the risk for T2D. The explosive increase in T2D prevalence in recent decades cannot be explained only by genetics, as it is unlikely that genomes changed during this relatively short period of time [34].

Some evidence suggests that DNA methylation may be an intermediate stage in T2D pathogenesis. When testing DNA methylation patterns as a potential contributor to variation in T2D risk across the genome, Hidalgo et al. identified a significant association of CpG methylation in two sites of the *ABCG1* gene with insulin levels and IR in CD4(+) T cells from normal subjects [41]. An association of CpG methylation in the thioredoxin-interacting protein (*TXNIP*) gene with T2D was recently observed. *TXNIP* is involved in glucose uptake by skeletal muscles and glucotoxicity-induced pancreatic β-cell apoptosis [34]. In addition to *TXNIP*, CpG loci of *ABCG1*, phosphoethanolamine/phosphocholine phosphatase *(PHOSPHO1)*, suppressor of cytokine signaling 3 *(SOCS3)* and *SREBF1* genes in peripheral blood were significantly associated with development of T2D [42]. Dayeh et al. assessed methylation in approximately 1649 CpG regions of 853 genes in pancreatic islet cells from 15 patients with T2D and 34 nondiabetic controls. They observed altered methylation profiles in all studied genes, including transcription factor 7 like 2 *(TCF7L2)*, fat mass and obesity-associated protein *(FTO)*, and kidney and cardiac voltage dependend K+ channel *[KCNQ1]*, as well as altered methylation and gene expression levels for 102 genes, including cyclin dependent kinase inhibitor 1A *(CDKN1A)*, phosphodiesterase 7B *(PDE7B)*, septin 9 *(SEPT9)*, and exocyst complex component 3 like 2 *(EXOC3L2)*. Importantly, methylation of these genes can affect pancreatic cell function [43].

The epigenetic mechanism underlying T2D pathogenesis remains poorly understood, although it is well established that environmental factors play a central role in T2D development and may modulate gene expression by epigenetic mechanisms [34]. Environmental factors can include the inadequate lifestyle associated with obesity pathogenesis, which has T2D as a commonly associated comorbidity. T2D is accompanied by altered metabolisms of methyl-, folic acid-, homocysteine-, and choline-donator cells. Homocysteine metabolism imbalance is an important biomarker for various diseases and can alter methyl group metabolism and epigenetic methylation control. Clinical studies associated hyperhomocysteinemia in diabetes with renal dysfunction and impaired ability to catabolize homocysteine as a result of disease progression [44]. T2D-induced changes in methyl group metabolism suggest that DNA methylation may be compromised, favoring hypomethylation.

DNA hypomethylation has been observed in T2D subjects. A large EWAS assessed the epigenome of peripheral blood from subjects (*n* = 1169) with T2D and control individuals [45]. Compared to controls, T2D subjects presented a greater number of differentially methylated sites in genomic loci previously associated with T2D in a GWAS [41]. Hypomethylation of CpG islands in the *FTO* gene (3.35%) was significantly associated with T2D risk. The authors suggested that the relationship between methylation and gene expression should be studied in tissues associated with T2D pathophysiology because *FTO* is expressed in many of these tissues (i.e., pancreatic islands, skeletal muscle, and adipose) [45].

Data from tissue methylation can provide new insights into the epigenetics of T2D. For instance, hypomethylation of genes from liver tissue seem to contribute to T2D pathogenesis. When comparing epigenetic changes of liver DNA in obese patients with (*n* = 35) and without (*n* = 60) T2D, Nilsson et al. found an association of T2D with 251 CpG regions with altered DNA methylation. These regions included CpG regions of T2D-related genes, such as growth factor receptor bound protein 10 *(GRB10)*, ATP binding cassette subfamily C member 3 *(ABCC3)*, monoacylglycerol O-acyltransferase 1 *(MOGAT1)*, and PR domain 16 *(PRDM16)*, approximately 94% of which were hypomethylated. Another 29 hepatic genes with altered methylation in T2D patients presented altered expression [46].

**Fig. 3:**
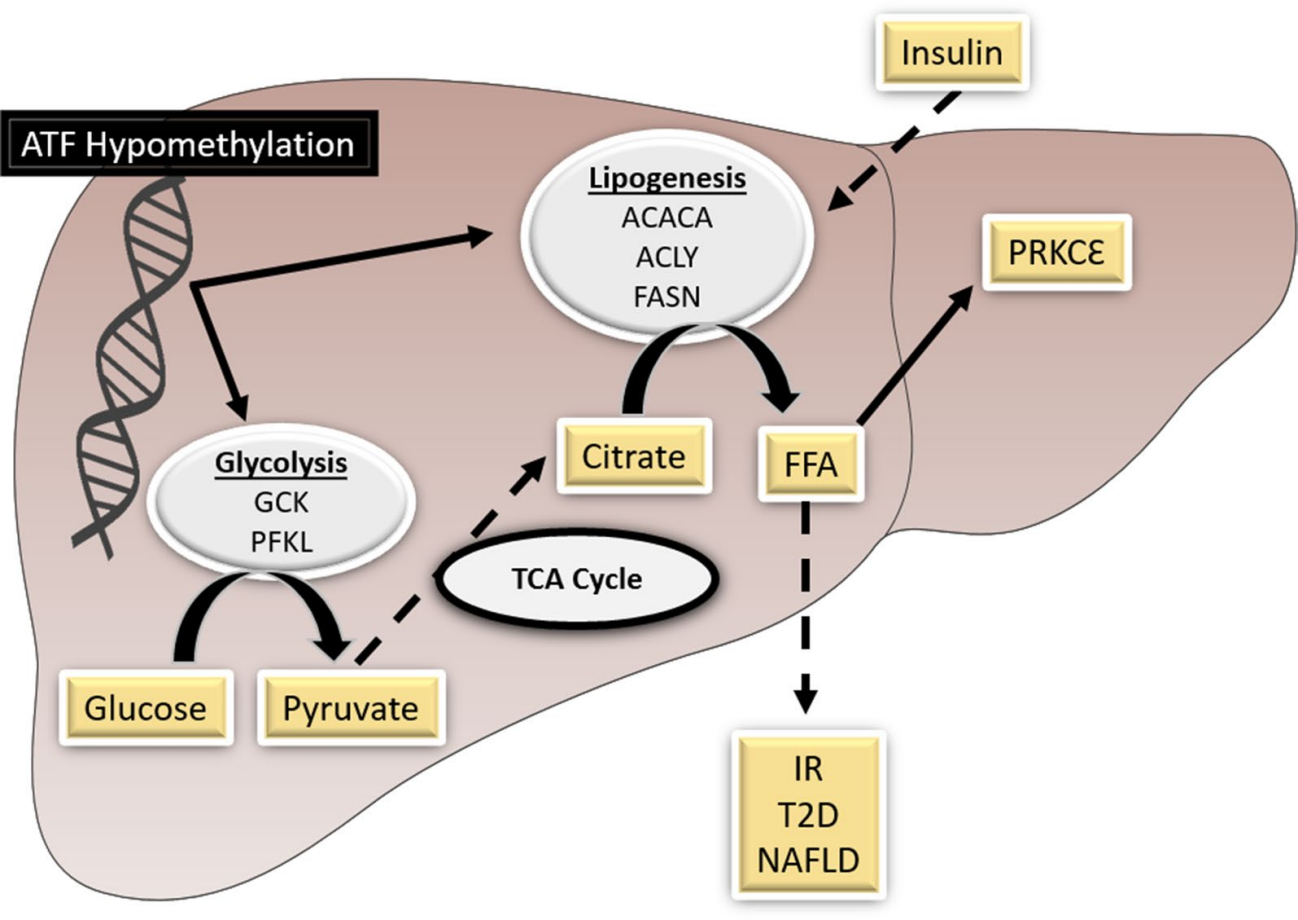
Proposed hypothesis of epigenetic mechanisms in liver contributing to IR in severe obesity, based on findings from Kirchner et al. [47]. Increased hepatic glycolysis and de novo lipogenesis are associated with DNA hypomethylation within ATF motifs of genes involved in glycolysis and IR. An excess of pyruvate from glycolysis is not used for ATP synthesis in the tricarboxylic acid (TCA) cycle and is converted to fatty free acids (FFA), which activate transcription of CƐ kinase that remains silenced by hypomethylation, increasing CE kinase (PRKCE) levels. The action and increased levels of PRKCE have been implicated in decreased insulin signaling. Therefore, the liver of severely obese patients is programmed to become insulin-resistant, possibly contributing to T2D and nonalcoholic fatty liver disease (NAFLD). *Continuous arrows* represent activation of studied glycolysis and lipogenesis pathways. *Discontinuous arrows* represent the proposed hypothesis

Kirchner et al. [47] evaluated global gene methylation and expression in hepatic tissues from severely obese men with or without T2D and from a nonobese control group. Severe obesity was associated with hypomethylation of genes involved in hepatic glucose metabolism and IR, in parallel with increased levels of gene expression. Binding sites for activating transcription factor (ATF) motifs were found in several of the genes, including glucokinase *(GCK)* and phosphofructokinase, liver type *(PFKL),* involved in liver glycolysis, acetyl-CoA carboxylase alpha *(ACACA),* ATP citrate lyase *(ACLY)*, fatty acid synthase (*FASN),* involved in de novo lipogenesis, and protein kinase C epsilon *(PRKCE),* involved in insulin signaling. Hypomethylation of CpG sites within ATF motifs in these genes was highlighted by a genome-wide DNA methylation assay of liver samples from severely obese patients with and without T2D, compared to nonobese controls. However, the DNA methylation pattern at these ATF motifs inversely mirrored the mRNA expression levels when comparing patients with and without T2D, except for *ACLY*. In addition, mRNA and protein expression levels of PRKCE were increased only in nondiabetic obese patients. The authors concluded that severe obesity is accompanied by changes in the methylation profiles of several genes controlling glucose metabolism within the ATF motif regulatory site. These changes are associated with *PRKCE* activation and hypomethylation and seem to favor liver glycolysis and lipogenesis, contributing to IR (Fig. 3).

In summary, acquired data support the association of epigenotypes with obesity and T2D risk. This information may aid in the identification of epigenetic markers with potential predictive value for early diagnosis of these diseases [6]. For instance, the predictive power of *FTO* methylation for T2D was independent of any known polymorphism and significantly higher than any genetic variant described to date. Other methylation markers of obesity and T2D, summarized in Table 1, may represent early markers for risk of obesity and T2D and the response to lifestyle, pharmacological, or surgical interventions [6].

**Table 1:** Summary of main studies of DNA methylation in obesity, T2D, and RYGB

Study design	Sample	Methylation sites and methods	Main results
Gene methylation: obesity			
Methylation of TNFα promoter in eutrophic women with decreased central adiposity (*n* = 40) [25]	White blood cells	Methylation of 20 CpG regions of *TNFα*, assessed by Sequenom EpiTyper MassARRAY	Women with low central adiposity showed greater methylation in 2 CpG regions, associated with lower BMI and % of body fat
Association between methylation profile and BMI in very young children (*n* = 120) [26]	Whole blood	Methylation of 10 CpG regions of *LEP*, assessed by Sequenom EpiTyper MassARRAY	An 0.8% decrease in *LEP* methylation levels was associated with an increase in BMI
Methylation status of 2 CpG regions of *POMC* gene in obese (*n* = 71) and normal-weight (*n* = 36) adolescents, with a validation cohort in children (*n* = 154) [27]	Whole blood	Methylation in 2 CpG regions of *POMC* gene, assessed by bisulfite sequencing	Lean adolescents had lower levels of *POMC* gene methylation
Epigenetic modifications in normal-weight (*n* = 20), overweight (*n* = 20), and obese women (*n* = 20) [28]	White blood cells	Methylation in CpG regions of *CLOCK*, *BMAL1*, and *PER2* genes, assessed by Sequenom EpiTyper MassARRAY	Lean women had lower levels of methylation in CpG regions at *BMAL1*. Methylations in multiple CpG regions of *CLOCK*, *BMAL1*, and *PER2* were positively associated with BMI, % of body fat, and waist circumference (WC)
Methylation profiles in obesity among monozygotic twins (*n* = 84) [30]	White blood cells	Methylation of 20 CpG regions of *SLC6A4*, assessed by pyrosequencing	An increase in *SLC6A4* gene methylation was associated with increases in BMI and WC
Methylation pattern and BMI in elderly men (*n* = 170) [31]	White blood cells	Methylation of 8 CpG regions of *AR*, assessed by Sequenom EpiTyper MassARRAY	Methylation of one region of CpG in the *AR* promoter was positively associated with BMI and % of body fat
Methylation profile and BMI in obese adults (*n* = 34) [32]	White blood cells	Methylation of *HSD2*, *GR*, *IGF2*, and *H19,* assessed by pyrosequencing	Methylation in several CpG regions in *HSD2*, *GR*, and *IGF2* was positively associated with WC and BMI
Methylation profiles of lean (*n* = 48) and obese (*n* = 48) adolescents [35]	White blood cells	Methylation of >450,000 CpG regions, assessed by Illumina Infinium Human Methylation 450 BeadChip	Differential methylation was observed in 23,305 CpG regions between lean and obese adolescents
Methylation profiles of lean (*n* = 24) and obese (*n* = 23) female preadolescents [36]	Whole blood	Methylation of >27,000 CpG regions, assessed by Illumina Infinium Human Methylation 27 BeadChip	There was a 15.5% difference in methylation of 20 CpG regions between the lean and obese groups
Genes methylation: T2D			
Methylation profiles in pancreatic islets from T2D (*n* = 15) and non-T2D (*n* = 34) donors [43]	Pancreatic islets	Gene methylation and expression in >450,000 CpG regions, assessed by Illumina Infinium Human Methylation 450 BeadChip and Affymetrix GeneChip Human Gene 1.0 ST array, respectively	In T2D islets, 853 genes (i.e., *TCF7L2*, *FTO*, and *KCNQ1*) showed altered methylation profiles, while 102 genes (e.g., *CDKN1A*, *PDE7B*, *SEPT9*, and *EXOC3L2*) showed altered gene methylation and expression profiles. Methylation of these genes can affect pancreatic cell function
Methylation profile in T2D (*n* = 710) and non-T2D patients (*n* = 459) [45]	Whole blood	Global methylation, assessed by Affymetrix SNP6 microarray	CpG region of *FTO* gene showed hypomethylation (*p* < 0.05) in T2D patients compared to control group
Methylation profile in T2D (*n* = 15), glucose-intolerant (*n* = 8), and non-T2D patients (*n* = 15) [51]	Skeletal muscle	Methylation and expression of *PPARG* and *PGC1A* genes, assessed by bisulfite sequencing and RT-qPCR, respectively	*PPARG* and *PGC1A* were hypermethylated in T2D patients compared to other groups. Methylation levels were negatively correlated with *PGC1A* gene expression
Epigenetic regulation of *PPARGC1A* in T2D (*n* = 48) and non-T2D (*n* = 12) islets [61]	Pancreatic islets	Methylation and expression of *PPARGC1A* gene, assessed by sequencing and RT-qPCR, respectively	*PPARGC1A* promoter showed increased methylation (*p* < 0.05) in T2D patients. *PPARGC1A* gene expression was reduced 90% (*p* < 0.005) and correlated with reduced insulin secretion in T2D patient islets
Methylation profiles in pancreatic islets from T2D (*n* = 9) and non-T2D (*n* = 48) donors [62]	Pancreatic islets	Methylation of 25 CpG regions of *INS* promoter assessed by Sequenom EpiTyper MassARRAY and pyrosequencing, and expression of *INS* assessed by RT-qPCR	*INS* promoter methylation was increased in T2D patients and negatively correlated with *INS* expression (*p* < 0.05)
*PDX1* methylation profiles in pancreatic islets from T2D (*n* = 9) and non-T2D (*n* = 55) donors [63]	Pancreatic islets	Methylation of 29 CpG regions of *PDX1* (15 CpG sites of the human distal *PDX1* promoter and the other assay covered 14 CpG sites of the human *PDX1* enhancer region) assessed by Sequenom EpiTyper MassARRAY and pyrosequencing, and expression of *PDX1* assessed by RT-qPCR	In T2D patient islets, 10 CpG regions of *PDX1* showed increased methylation compared to non-T2D islets. *PDX1* methylation was negatively correlated with gene expression
*MCP1* methylation profiles in T2D (*n* = 32) and non-T2D patients (*n* = 15) [64]	White blood cells	Methylation of *MCP1* gene, assessed by PCR	*MCP1* promoter region was methylated in the control group. *MCP1* showed less methylation (*p* < 0.001) but higher serum levels in T2D patients
Genes methylation: RYGB			
Obese T2D women with weight loss after RYGB (*n* = 8) compared to non-obese women (*n* = 9) [29]	Skeletal muscle	Methylation of *PGC1A* and *PDK4* promoters, assessed by bisulfite sequencing	*PGC1A* and *PDK4* promoter methylation levels were modified by obesity and restored to normal levels after weight loss induced by RYGB
Folate levels and epigenetic alterations in liver from subjects with T2D [46]	Liver	Methylation and gene expression of >450,000 CpG regions, assessed by Illumina Infinium Human Methylation 450 BeadChip and RT-qPCR, respectively	In T2D patients, 251 CpG regions, including regions in *GRB10*, *ABCC3*, *MOGAT1*, and *PRDM16*, exhibited altered methylation compared to nondiabetic subjects. About 94% of modified CpG regions showed a decrease in hepatic DNA methylation in T2D patients. Hypomethylation was correlated with lower folate levels. T2D patients showed decreased erythrocyte folate levels compared to nondiabetic patients (*p* < 0.05). Finally, 29 other hepatic genes showed alterations in methylation and gene expression in T2D patients
DNA methylation and hydroxymethylationin relation to energy-restricted diet (*n* = 22) or bariatric surgery (*n* = 14) [48]	Whole blood	Methylation assessed by PCR	Baseline *LINE*-*1* methylation was associated with serum glucose levels. Baseline hydroxymethylation was associated with BMI, WC, total cholesterol, and triglyceride levels. *LINE*-*1* and *SERPINE*-*1* methylation levels did not change after weight loss. *IL6* methylation increased after energy restriction and decreased after bariatric surgery. *SERPINE*-*1* methylation was associated with weight loss response
Promoter methylation after RYGB and VLCD in obese patients (*n* = 18) [49]	Whole blood	Methylation of promoter regions of *PPARGC1A, PDK4, TFAM, IL1B, IL6*, and *TNFα* genes, assessed by PCR	VLCD decreased methylation of *PPARGC1A*. Methylation levels of *PPARGC1A, IL1B, IL6,* and *TNFα* promoters were decreased 2 days after RYGB, but methylation levels of *PDK4, IL1B, IL6,* and *TNFα* promoters were increased 12 months after RYGB (*p* < 0.05)
Methylation in obese patients (*n* = 11) 6 months after RYGB compared to health people (*n* = 16) [52]	Whole blood	Methylation of >450,000 CpG regions, assessed by Illumina Infinium Human Methylation 450 BeadChip	*ADK* gene methylation was 10% lower 6 months after RYGB. *ADK* encodes adenosine kinase, a potential regulator of extracellular adenosine and intracellular nucleotide (e.g., adenina) levels. Adenosine can improve insulin secretion and decrease glucose production
Differential methylation in obesity and T2D genes in siblings born before (*n* = 531) and after (*n* = 531) maternal bariatric surgery [53]	Whole blood	Methylation of >450,000 CpG regions, assessed by Illumina Infinium Human Methylation 450 BeadChip	Comparing siblings born before vs. after surgery, 3074 genes were differentially methylated, with an overrepresentation of genes involved in insulin receptor, T2D, and leptin signaling in obesity. *HLA*-*DQA1, HLA*-*DQB1*, and *TSPAN18* were the most significantly differentially methylated genes
DNA methylation analysis in obese patients with NAFLD before (*n* = 45) and after bariatric surgery (*n* = 23) [54]	Liver	Methylation and gene expression of >450.,000 CpG regions, assessed by Illumina Infinium Human Methylation 450 BeadChip and Affymetrix Human Gene 1.1 ST, respectively	Gene ontology and transcription factor binding site analyses revealed distinct postsurgery and NAFLD-specific methylation signatures, with >400-fold enrichment of *NRF1*, *HSF1*, and *ESRRA* sites. The findings illustrate treatment-induced epigenetic liver remodeling. *PTPRE* showed hypermethylation and decreased gene expression after RYGB. This result may represent a key mechanism for reestablishment of hepatic insulin sensitivity during weight loss
DNA methylation in adipose tissue from obese women (*n* = 15) before and after weight loss by gastric bypass [55]	Subcutaneous and omental adipose tissue	Gene methylation and expression assessed for >450,000 CpG regions, by the Illumina Infinium Human Methylation 450 BeadChip and RT-qPCR, respectively	Differential methylation was observed in omental and subcutaneous adipose tissue (*p* < 0.05). A greater proportion of CpG regions were hypermethylated before weight loss. Increased methylation was observed in the 3′ untranslated region and gene bodies relative to promoter regions. Differential methylation was found within genes associated with obesity, epigenetic regulation, and development, such as *CETP, FOXP2, HDAC4, DNMT3B, KCNQ1*, and *HOX* clusters. Robust correlations were observed between changes in methylation and clinical traits, including associations between fasting glucose and *HDAC4, SLC37A3*, and *DENND1C* in subcutaneous adipose. Genes investigated with differential promoter methylation all showed significantly different levels of mRNA before and after gastric bypass
Fat cell epigenetic signature in women 2 years after RYGB (*n* = 16) compared to lean women (*n* = 14) [56]	Subcutaneous adipose tissue	Methylation of >450,000 CpG regions and gene expression, assessed by Illumina Infinium Human Methylation 450 BeadChip and Human Gene 1.1 ST, respectively	After RYGB, 8504 CpG regions showed methylation in adipose tissue. After RYGB, 3717 genes were overexpressed and associated with cell differentiation pathways. Among the adipogenesis-associated genes, 27% presented altered methylation in patients after RYGB compared to the control group
Longitudinal genome-wide methylation study in obese patients (*n* = 11) with hypertension 6 months after RYGB [59]	Whole blood	Methylation of >450,000 CpG regions, assessed by Illumina Infinium Human Methylation 450 BeadChip	There were 24 promoters associated with CpG regions. Data were correlated with systolic blood pressure changes after RYGB. Two CpG loci (cg00875989, cg09134341) were hypomethylated and associated with hypertension

### Epigenetic response to bariatric surgery

Conventional strategies for the treatment of obesity and its comorbidities (i.e., lifestyle changes) often have poor long-term adherence, and pharmacological interventions are limited [29]. Bariatric surgery is the most effective current option to treat severe obesity, and RYGB is the most applied technique worldwide. Considered a “metabolic surgery”, RYGB increases insulin sensibility even before substantial weight loss is achieved, contributing to clinical improvement or remission of T2D.

Although early alterations in intestinal hormone (i.e., incretin) release have been suggested as a potential factor involved in post-RYGB glucose homeostasis, the molecular mechanisms associated with these alterations are poorly understood [29]. Similarly to physical activity and dietetic interventions, bariatric surgery can change DNA methylation patterns in different biological samples [2]. However, the epigenetic changes differ depending on the approach used to treat obesity and its associated comorbidities (clinical or surgical).

Nicoletti et al. studied whether induced weight loss can affect global DNA methylation of long interspersed nuclear elements (e.g., *LINE*-*1*) or the methylation and hydroxymethylation profiles (as markers of increased gene expression) of inflammatory genes in obese patients. Their study focused on 22 obese patients who underwent caloric restriction based on a Mediterranean diet, 14 obese patients who underwent bariatric surgery, and 9 nonobese controls. DNA methylation patterns depended on the applied obesity treatment. *LINE*-*1* methylation levels did not change after weight loss, regardless of treatment, whereas interleukin 6 (*IL6*) methylation increased after energy restriction and decreased after bariatric surgery [48].

Another study considered the methylation profiles of promoters of several genes (PPARG coactivator 1 alpha *[PPARGC1A]*, *PDK4*, transcription factor A, mitochondrial *[TFAM]*, interleukin 1 beta *[IL1B]*, *IL6*, and *TNF*) in blood from obese and nonobese patients. This study included severely obese nondiabetic patients who were on a very low calorie diet (VLCD) for 14 days (*n* = 18), followed in some cases by RYGB (*n* = 7), and nonobese control patients who underwent elective cholecystectomy (*n* = 6) [49]. Decreased methylation in the promoter of *PPARGC1A* was observed after VLDC. Significantly decreased levels of methylation were observed in the promoters of *PPARGC1A*, *IL1B*, *IL6*, and *TNF* 2 days after RYGB. However, significantly increased methylation levels were observed in the promoters of *PDK4*, *IL1B*, *IL6*, and *TNF* 12 months after RYGB. These data suggest that epigenetic changes after intervention for obesity differ according to the intervention type. Compared to clinical approaches, epigenetic changes seem to be more relevant after bariatric surgery and potentially contribute to postoperative metabolic homeostasis [49].

Compared to primary clinical care, bariatric surgery leads to much greater loss of body weight and higher remission rates of T2D and metabolic syndrome [50], with methylation profiles in promoter regions of genes in obese individuals becoming similar to those of normal-weight individuals [2]. Barres et al. compared DNA methylation and gene expression levels in skeletal muscle of nonobese control women (*n* = 9) to levels in obese women (*n* = 8) before and 6 months after RYGB. Obesity was associated with changes in expression levels of genes related to mitochondrial function. After bariatric surgery, expression levels of 11 of the 14 studied genes were similar to those of nonobese control patients. Methylation of promoter regions of *PGC1A* and *PDK4* were altered in obese women, but normalized after RYGB to the levels in control women [29]. Normalization of gene expression and methylation occurred in parallel with normalization of the fasting glucose, insulin, total cholesterol, low-density lipoprotein, triglyceride, and nonesterified fatty acid levels after RYGB surgery. The authors observed an opposite pattern between DNA methylation and mRNA expression of *PGC1A*, suggesting that DNA methylation is involved in mRNA expression [50].

Methylation of a single cytosine residue in the *PGC1A* promoter induced a marked reduction in gene activity [51]. Obesity was associated with hypermethylation of CpG and exonic regions near transcription initiation sites, as well as methylation of a single cytosine residue into a non-CpG site in the *PDK4* promoter, which decreased gene activity. These findings suggest that altered DNA methylation, even in non-CpG regions, is involved in the physiological control of gene transcription in obese women. RYGB can normalize these altered methylation patterns in parallel with improvements in clinical variables associated with insulin sensitivity after weight loss [29].

In another study, the response of DNA methylation in promoter regions to RYGB was assessed in whole blood from obese patients before and 6 months after RYGB (*n* = 11) and nonobese controls (*n* = 16), in parallel with fasting glucose levels. RYGB was associated with changes in DNA methylation in 51 promoters (28 positively and 23 negatively regulated), with enrichment for genes involved in metabolic processes. In contrast to data before surgery, the mean DNA methylation level in all of these promoters after RYGB was similar to that found in the control group. When the authors controlled for weight loss (−24%) and fasting glucose (−16%) after RYGB, only the promoter for adenosine kinase *(ADK)* presented with DNA methylation significantly different from preoperative values (10% lower than basal). Adenosine can improve insulin secretion, decrease glucose production, and stimulate glucagon secretion [52]. These data suggest that changes in DNA methylation after RYGB may contribute to the observed improvement in glucose metabolism. These data also support an important role for epigenetic changes in development and predisposition to metabolic diseases, such as obesity and T2D [52].

Epigenetic changes in genes involved in obesity and glucose homeostasis experienced by obese women after bariatric surgery may be inherited by their descendants [53]. A recent retrospective study examined DNA methylation patterns in the blood of brothers born before (*n* = 531) and after (*n* = 531) maternal bariatric surgery. Siblings born before versus after bariatric surgery showed different methylation patterns in 3074 genes involved in insulin receptor signaling, T2D, leptin signaling, and obesity [53].

Organ-specific epigenetic signatures after bariatric surgery have been identified in organs involved in obesity and T2D pathogenesis. Ahrens et al. obtained liver biopsies from 63 patients, classified histologically as normal controls (*n* = 18) and nonalcoholic fatty liver disease (NAFLD) patients distributed into healthy obese (*n* = 18), steatosis (*n* = 12), and nonalcoholic steatohepatitis (NASH, *n* = 15) phenotypes [54]. Altered expression and methylation patterns of nine genes encoding key enzymes involved in intermediary metabolism (e.g., pyruvate carboxylase *[PC]*, *ACLY*, and phospholipase C gamma 1 *[PLCG1]*) and insulin signaling (e.g., insulin like growth factor 1 *[IGF1]*, insulin like growth factor binding protein 2 *[IGFBP2]*, and *PRKCE*) were associated with NAFLD. When the analysis was replicated in 39 independent liver samples from NAFLD obese patients, high transcription levels in CpG regions of zinc finger protein 274 *(ZNF274)*, *PGC1A*, and sterol regulatory element binding transcription factor 2 *(SREBP2)* were associated with liver disease [54]. Some of these obese patients (*n* = 23) underwent bariatric surgery and were reevaluated 5–9 months after this procedure. Despite the expected improvement of liver histology, the intraindividual comparison of liver biopsies before and after bariatric surgery highlighted methylation changes associated with liver disease, which appeared to be partially reversible by the surgery. An inverse correlation of the NASH phenotype and bariatric surgery was observed, indicating that methylation changes associated with NASH can be reversed after surgery. Specific methylation signatures of NAFLD-related genes (i.e., nuclear respiratory factor 1 *[NRF1]*, heat shock transcription factor 1 *[HSF1]*, and estrogen related receptor alpha *[ESRRA]*) were clearly distinct after the bariatric procedure, both in gene ontology and transcription factor binding site analyses. The receptor protein tyrosine phosphatase epsilon (PTPRE)-encoding gene exhibited altered expression and methylation patterns before and after surgery. Hypermethylation and low expression of *PTPRE* are negative regulators of insulin signaling in skeletal muscle, suggesting that changes in its expression and methylation after bariatric surgery may be involved in the hepatic insulin sensitivity restoration observed after the procedure [54].

Adipose tissue plays a key role in the metabolic disorder related to obesity. Another study on organ-specific epigenetic signatures after bariatric surgery assessed DNA methylation profiles in 15 obese women before and a mean of 17.5 months after RYGB, when patients had achieved up to 27% of body weight loss [55]. After RYGB, significant differences in obesity-related gene methylation levels were found in subcutaneous and omental adipose tissues. Most CpG islands were hypermethylated before weight loss. Changes in methylation levels of genes, including histone deacetylase 4 *(HDAC4)*, solute carrier family 37 member 3 *(SLC37A3)*, and DENN domain containing 1 *(DENND1C),* also known as FAM31C, in subcutaneous adipose tissue, correlated with fasting glycemia levels. Significantly different mRNA levels of all studied genes presenting with methylation in their promoter regions were found when comparing before and after RYGB. These findings suggest that altered DNA methylation of adipose tissue may have a role in obesity development and can be changed by RYGB, potentially contributing to glucose homeostasis [55].

Despite the benefits associated with metabolic and weight loss responses to bariatric surgeries, patients who undergo these procedures still exhibit a higher risk of chronic diseases (including T2D relapse) and mortality compared to the general population [56]. Postobese patients have fat cells that are smaller in size and greater in number compared to never-obese subjects [57]. Such metabolic disturbances of the fat cells may contribute to postoperative complications [58].

A clinical study tested the hypothesis that differentially methylated DNA sites are associated with adipose tissue hyperplasia after bariatric surgery-induced weight loss. Genome-wide DNA methylation was analyzed in abdominal subcutaneous fat cells collected from 16 women 2 years after gastric bypass surgery with significant weight loss (BMI 26 ± 2 kg/m^2^) and from 14 never-obese women (BMI 25 ± 2 kg/m^2^), both presenting stable weight for the previous 6 months. Gene expression was assessed in the adipose tissues of 9 women from each group. Compared to the never-obese group, the bariatric surgery group showed significant global DNA hypomethylation and a greater number of differentially methylated sites in adipogenesis genes. In a secondary analysis, the authors assessed DNA methylation and expression of adipogenesis genes in 15 and 11 obese women, respectively. Differential methylation of adipogenesis genes was accompanied by altered expression levels in obese but not postobese women. The authors concluded that altered methylation of adipogenesis genes in fat cells can contribute to adipose tissue hyperplasia in women after bariatric surgery [56].

Data obtained until now support a remodeling response of altered gene methylation to bariatric surgery, with potential effects favoring metabolic homeostasis. These effects may include remission of T2D as a comorbidity of obesity. Table 1 summarizes the main studies involving DNA methylation in obesity, T2D, and RYGB. Other clinical benefits associated with bariatric surgery seem to involve epigenetic changes. Twenty-four promoters associated with CpG loci were correlated with changes in blood pressure after RYGB. Two of these promoters were significantly hypomethylated depending on the presence of hypertension [59]. Nevertheless, interventional bariatric surgeries are not free of complications that may also involve epigenetic changes.

Current findings are aiding in the identification of epigenetic markers with potential predictive value for clinical responses induced by the surgical procedure [6]. For instance, baseline methylation levels of serpin family E member 1 *(SERPINE*-*1)* were significantly lower in obese patients presenting relevant weight loss after bariatric surgery. Mechanisms dependent on the inhibitor of plasminogen activator (PAI-1) encoded by *SERPINE*-*1* are involved in the pathogenesis of obesity, IR, and T2D [60], and methylation levels of *SERPINE*-*1* have been associated with obesity and metabolic syndrome [48]. Therefore, *SERPINE*-*1* gene methylation may be a predictive marker of weight loss response to RYGB [48].

## Final considerations and conclusions

Thanks to advances in next-generation technologies, the field of epigenetics is growing quickly. Not long ago, researchers believed that DNA methylation represented only a gene-silencing mark, and that its increase in promoter regions was associated with decreased gene expression. Recent studies suggest that DNA methylation also affects noncoding RNA expression, transcriptional elongation, splicing events, and overall genomic stability, depending on the genomic location in CpG regions where methylation occurs [46]. Some of these epigenetic changes have been associated with the development of obesity and T2D. DNA methylation analysis of the entire genome is opening a new window for identifying phenotypes and epigenetic signatures of these clinical conditions [2]. Several methods are commercially available to assess global DNA methylation, with the Illumina Infinium Human Methylation 450 BeadChip^®^ emerging as the most widely used platform for this purpose. This method analyzes more than 450,000 methylation regions in CpG sites and other nearby regions, providing reproducible quantitative data that could facilitate comparisons across studies [2].

Epigenetic researchers in the areas of obesity and T2D seek to identify biomarkers that can predict the risk of developing these diseases and their associated complications. They aim to understand the environmental factors related to obesity, which can modulate gene expression by affecting epigenetic mechanisms. Such understandings would allow the design of new therapeutic strategies. However, the characterization of all of the factors able to modify epigenetic signatures and their true importance for obesity and T2D are affected by the small and cumulative magnitude of changes produced by dietary and environmental factors, which differ largely between biological samples [44].

Peripheral blood is the DNA source most applied in epigenetic studies because of the ease of analysis [2]. However, blood contains a mixture of cell types with different methylation profiles, such that its use in epigenetic studies may not necessarily reflect the epigenetic status of other tissues. Studies that provide the “human epigenetic profile” will be of considerable value to identify tissue-specific epigenetic signatures and their role in the development of chronic diseases [2]. In humans, the analysis of epigenetic profiles showed significant differences between normal weight and obese individuals, and between T2D individuals and nondiabetic controls [6]. These differences were mainly observed in peripheral blood, but also in other types of cells (i.e., pancreatic islets) and tissues (i.e., muscle, liver and adipose tissue). These and additional findings may help in elucidating why obesity and T2D develop in some individuals but not others.

Recent findings suggest that epigenetic changes in specific tissues of patients with T2D are at least partially co-regulated by variations in DNA sequence. Body weight reduction can have a dynamic effect on the epigenome. Considering the role of obesity in T2D development, the epigenome is an innovative target for pharmacological and environmental therapeutic interventions [6]. The identification of predictive epigenetic biomarkers for obesity and remodeling markers induced by bariatric surgery may be useful to achieve this aim.

Potential biomarkers for obesity can be detected early, enabling prediction of disease risk at a young age, before the phenotype develops. This observation opens the promising possibility to design intervention strategies for the prevention of obesity and, consequently, T2D. Bariatric surgery can influence DNA methylation in parallel with changes in gene expression pattern. Weight loss that can be achieved by these procedures is associated with changes in the methylation of CpG sites and between exon regions near the transcription sites. Dynamic changes in DNA methylation might be an early event that drives the transcription of genes involved in the orchestral regulation of insulin sensitivity in human obesity [29].

Epigenetic regulation seems to be tissue specific; therefore, it is important to investigate a homogeneous cell type or tissue from a target organ for development of the studied disease. The pancreatic islet is one of the most thoroughly investigated cell types, and the liver, muscle, and adipose tissues are the main investigated tissues for T2D. Changes in clinical biomarkers that reflect improvements in glucose and lipid metabolism after RYGB often occur before major weight loss and are coordinated by surgery-induced changes in intestinal hormones. Therefore, the intestine has been the subject of major studies on T2D and bariatric surgery [65, 66]. The intestinal methylation profile would assist in understanding the mechanisms involved in improved glycemic control after bariatric surgery. To complement studies with tissue-specific approaches to assess the epigenetics of T2D, we are currently exploring the effects of bariatric surgery on the gastrointestinal methylation profile of obese patients with T2D, as part of the SURMetaGIT study (SURgically induced Metabolic effects on the Human GastroIntestinal Tract) [67].

In conclusion, evidence supports a potential role of epigenetic changes in obesity and T2D pathogenesis. Epigenetic profiles seem to be altered by bariatric surgeries towards metabolic homeostasis. The main objectives in this area for the future are to identify epigenetic marks that could be used as early indicators of metabolic risk, and to develop treatments able to delay or even reverse these epigenetic changes. Further studies should apply methods based on global analysis of the genome (preferentially in disease-target tissues) to identify methylated sites associated with disease and epigenetic marks associated with the remodeling response to bariatric surgery.

## Abbreviations

ABCC3: ATP binding cassette subfamily C member 3
ABCG1: ATP binding cassette subfamily G member 1
ACACA: acetyl-CoA carboxylase alpha
ACLY: ATP citrate lyase
ADK: adenosine kinase
ADRB3: adrenoceptor beta 3
AR: androgen receptor
ATF: activating transcription factor
BMAL1: aryl hydrocarbon receptor nuclear translocator-1
BMI: body mass index
CDKN1A: cyclin dependent kinase inhibitor 1A
CETP: cholesteryl ester transfer protein
CLOCK: clock circadian regulator
CpG: cytosine-phosphate-guanine
CPT1A: carnitine palmitoyltransferase 1A
DENND1C: DENN domain containing 1 (also known as FAM31C)
DNMTs: DNA methyltransferases
ESRRA: estrogen related receptor alpha
EXOC3L2: exocyst complex component 3 like 2
EWAS: epigenome-wide association studies
FASN: fatty acid synthase
FFA: fatty free acids
FOXP2: forkhead box P2
FTO: fat mass and obesity-associated protein
GCK: glucokinase
GR: glucocorticoid receptor
GRB10: growth factor receptor bound protein 10
GWAS: genome-wide association study
HDAC4: histone deacetylase 4
HIF3A: hypoxia inducible fator 3 alpha subunit
HLA-DQA1: major histocompatibility complex, class II, DQ alpha 1
HLA-DQB1: major histocompatibility complex, class II, DQ beta 1
HOX: homeobox
HSD2: 11-beta-hydroxysteroid dehydrogenase type 2
HSF1: heat shock transcription factor 1
H19: H19, imprinted maternally expressed transcript
IGF1: insulin like growth factor 1
IGF2: insulin like growth factor 2
IGFBP2: insulin like growth factor binding protein 2
IL1B: interleukin 1 beta
IL6: interleukin 6
INS: insulin
IR: insulin resistance
KCNQ1: kidney and cardiac voltage dependend K+ channel
LEP: leptina
LINE-1: long interspersed nuclear elements
LY86: lymphocyte antigen 86
MCHR1: type 1 receptor for melanin-concentrating hormone
MCP1: monocyte chemoattractant protein-1
MOGAT1: monoacylglycerol O-acyltransferase 1
NAFLD: nonalcoholic fatty liver disease
NASH: nonalcoholic steatohepatitis
NRF1: nuclear respiratory factor 1
PAI-1: inhibitor of plasminogen activator
PC: pyruvate carboxylase
PCR: polymerase chain reaction
PDE7B: phosphodiesterase 7B
PDK4: pyruvate dehydrogenase kinase 4
PDX1: pancreatic and duodenal homeobox 1
PER2: period circadian clock 2
PFKL: phosphofructokinase, liver type
PGC1A: proliferator-activated receptor G coactivator 1A
PHOSPHO1: phosphoethanolamine/phosphocholine phosphatase
PLCG1: phospholipase C gamma 1
POMC: proopiomelanocortin
PPARG: peroxisome proliferator activated receptor gamma
PPARGC1A: PPARG coactivator 1 alpha
PRDM16: PR domain 16
PRKCE: protein kinase C epsilon
PTPRE: receptor protein tyrosine phosphatase épsilon
RT-qPCR: real-time quantitative polymerase chain reaction
RYGB: Roux-en-Y gastric by-pass
SEPT9: septin 9
SERPINE-1: serpin family E member 1
SREBP2: sterol regulatory element binding transcription factor 2
SLC6A4: serotonin transporter
SLC37A3: solute carrier family 37 member 3
SNPs: single nucleotide polymorphisms
SOCS3: suppressor of cytokine signaling 3
SREBF1: sterol regulatory element binding transcription factor 1
SURMetaGIT: SURgically induced Metabolic effects on the Human GastroIntestinal Tract
TCA: tricarboxylic acid cycle
TCF7L2: transcription factor 7 like 2
TDG: thymine DNA glycosylase
TET: ten-eleven translocation
TFAM: transcription factor A, mitochondrial
TNF: tumor necrosis fator
TNFα: tumor necrosis fator alpha
TSPAN18: tetraspanin 18
TXNIP: thioredoxin-interacting protein
T2D: type 2 diabetes
VLCD: very low calorie diet
WC: waist circumference
ZNF274: zinc finger protein 274
5caC: 5-carboxylcytosine
5FC: 5-formylcytosine
5hmC: 5-hydroxymethylcytosine
5mC: 5-methylcytosine

## References

[1] Baylin SB, Jones PA (2011). A decade of exploring the cancer epigenome: biological and translational implications. Nat Rev Cancer.

[2] van Dijk SJ, Molloy PL, Varinli H, Morrison JL, Muhlhausler BS, Members of EpiSCOPE (2015). Epigenetics and human obesity. Int J Obes.

[3] Li J, Harris RA, Cheung SW, Coarfa C, Jeong M, Goodell MA (2012). Genomic hypomethylation in the human germline associates with selective structural mutability in the human genome. PLoS Genet.

[4] Ahuja N, Easwaran H, Baylin SB (2014). Harnessing the potential of epigenetic therapy to target solid tumors. J Clin Invest.

[5] Lewin B (2009). Genes IX.

[6] Raciti GA, Longo M, Parrillo L (2015). Understanding type 2 diabetes: from genetics to epigenetics. Acta Diabetol.

[7] Bird A (2002). DNA methylation patterns and epigenetic memory. Genes Dev.

[8] Bernstein BE, Meissner A, Lander ES (2007). The mammalian epigenome. Cell.

[9] Shen L, Zhang Y (2012). Enzymatic analysis of Tet proteins: key enzymes in the metabolism of DNA methylation. Methods Enzymol.

[10] He YF, Li BZ, Li Z, Liu P, Wang Y, Tang Q (2011). Tet-mediated formation of 5-carboxylcytosine and its excision by TDG in mammalian DNA. Science.

[11] Ito S, Shen L, Dai Q, Wu SC, Collins LB, Swenberg JA (2011). Tet proteins can convert 5-methylcytosine to 5-formylcytosine and 5-carboxylcytosine. Science.

[12] Lister R, Pelizzola M, Dowen RH, Hawkins RD, Hon G, Tonti-Filippini J (2009). Human DNA methylomes at base resolution show widespread epigenomic differences. Nature.

[13] Suzuki MM, Bird A (2008). DNA methylation landscapes: provocative insights from epigenomics. Nat Rev Genet.

[14] Riggs AD (1975). X inactivation, differentiation, and DNA methylation. Cytogenet Cell Genet.

[15] Nguyen CT, Gonzales FA, Jones PA (2001). Altered chromatin structure associated with methylation-induced gene silencing in cancer cells: correlation of accessibility, methylation, MeCP2 binding and acetylation. Nucleic Acids Res.

[16] Yan J, Zierath JR, Barrès R (2011). Evidence for non-CpG methylation in mammals. Exp Cell Res.

[17] Dayeh TA, Olsson AH, Volkov P, Almgren P, Rönn T, Ling C (2013). Identification of CpG-SNPs associated with type 2 diabetes and differential DNA methylation in human pancreatic islets. Diabetologia.

[18] Rideout WM, Coetzee GA, Olumi AF, Jones PA (1990). 5-Methylcytosine as an endogenous mutagen in the human LDL receptor and p53 genes. Science.

[19] Kao SH, Wu KJ, Lee WH (2016). Hypoxia, epithelial-mesenchymal transition, and TET-mediated epigenetic changes. J Clin Med.

[20] Raciti GA, Beguinot F. Epigenetics of T2DM. Diapedia. http://www.diapedia.org/3105513816/rev/3. Accessed 09 Feb 2015.

[21] Billings LK, Florez JC (2010). The genetics of type 2 diabetes: what have we learned from GWAS?. Ann NY Acad Sci.

[22] Raciti GA, Nigro C, Longo M, Parrillo L, Miele C, Formisano P (2014). Personalized medicine and type 2 diabetes: lesson from epigenetics. Epigenomics.

[23] Feinberg AP, Irizarry R, Fradin D, Aryee MJ, Murakami P, Aspelund T (2010). Personalized epigenomic signatures that are stable over time and covary with body mass index. Sci Transl Med.

[24] Drong AW, Nicholson G, Hedman AK, Meduri E, Grundberg E, Small KS (2013). The presence of methylation quantitative trait loci indicates a direct genetic influence on the level of DNA methylation in adipose tissue. PLoS ONE.

[25] Hermsdorff HH, Mansego ML, Campión J, Milagro FI, Zulet MA, Martínez JA (2013). TNFalpha promoter methylation in peripheral white blood cells: relationship with circulating TNFα, truncal fat and n-6 PUFA intake in young women. Cytokine.

[26] Obermann-Borst SA, Eilers PHC, Tobi EW, de Jong FH, Slagboom PE, Heijmans BT (2013). Duration of breastfeeding and gender are associated with methylation of the LEPTIN gene in very young children. Pediatr Res.

[27] Kuehnen P, Mischke M, Wiegand S, Sers C, Horsthemke B, Lau S (2012). An Alu element-associated hypermethylation variant of the *POMC* gene is associated with childhood obesity. PLoS Genet.

[28] Milagro FI, Gómez-Abellán P, Campión J, Martínez JA, Ordovás JM, Garaulet M (2012). CLOCK, PER2 and BMAL1 DNA methylation: association with obesity and metabolic syndrome characteristics and monounsaturated fat intake. Chronobiol Int.

[29] Barres R, Kirchner H, Rasmussen M, Yan J, Kantor FR, Krook A (2013). Weight loss after gastric bypass surgery in human obesity remodels promoter methylation. Cell Rep.

[30] Zhao J, Goldberg J, Vaccarino V (2013). Promoter methylation of serotonin transporter gene is associated with obesity measures: a monozygotic twin study. Int J Obes.

[31] Movérare-Skrtic S, Mellström D, Vandenput L, Ehrich M, Ohlsson C (2009). Peripheral blood leukocyte distribution and body mass index are associated with the methylation pattern of the androgen receptor promoter. Endocrine.

[32] Drake AJ, McPherson RC, Godfrey KM, Cooper C, Lillycrop K, Hanson M (2012). An unbalanced maternal diet in pregnancy associates with offspring epigenetic changes in genes controlling glucocorticoid action and foetal growth. Clin Endocrinol.

[33] Stepanow S, Reichwald K, Huse K, Gausmann U, Nebel A, Rosenstiel P (2011). Allele-specific, age-dependent and BMI-associated DNA methylation of human MCHR1. PLoS ONE.

[34] Kwak SH, Park KS (2016). Recent progress in genetic and epigenetic research on type 2 diabetes. Exp Mol Med.

[35] Xu X, Su S, Barnes V, De Miguel C, Pollock J, Ownby D (2013). A genome-wide methylation study on obesity: differential variability and differential methylation. Epigenetics.

[36] Almén MS, Jacobsson J, Moschonis G, Benedict C, Chrousos GP, Fredriksson R (2012). Genome wide analysis reveals association of a FTO gene variant with epigenetic changes. Genomics.

[37] van Dijk SJ, Tellam RL, Morrison JL, Muhlhausler BS, Molloy PL (2015). Recent developments on the role of epigenetics in obesity and metabolic disease. Clin Epigenet.

[38] Hemminki K, Li X, Sundquist K, Sundquist J (2010). Familial risks for type 2 diabetes in Sweden. Diabetes Care.

[39] Meigs JB, Cupples LA, Wilson PW (2000). Parental transmission of type 2 diabetes: the Framingham Offspring Study. Diabetes.

[40] Sladek R, Rocheleau G, Rung J, Dina C, Shen L, Serre D (2007). A genome-wide association study identifies novel risk loci for type 2 diabetes. Nature.

[41] Hidalgo B, Irvin MR, Sha J, Zhi D, Aslibekyan S, Absher D (2014). Epigenome-wide association study of fasting measures of glucose, insulin, and HOMA-IR in the Genetics of Lipid Lowering Drugs and Diet Network Study. Diabetes.

[42] Chambers JC, Loh M, Lehne B, Drong A, Kriebel J, Motta V (2015). Epigenome-wide association of DNA methylation markers in peripheral blood from Indian Asians and Europeans with incident type 2 diabetes: a nested case-control study. Lancet Diabetes Endocrinol.

[43] Dayeh T, Volkov P, Salö S, Hall E, Nilsson E, Olsson AH (2014). Genome-wide DNA methylation analysis of human pancreatic islets from type 2 diabetic and non-diabetic donors identifies candidate genes that influence insulin secretion. PLoS Genet.

[44] Martínez JA, Milagro FI, Claycombe KJ, Schalinske KL (2014). Epigenetics in adipose tissue, obesity, weight loss, and diabetes. Adv Nutr.

[45] Toperoff G, Aran D, Kark JD (2012). Genome-wide survey reveals predisposing diabetes type 2-related DNA methylation variations in human peripheral blood. Hum Mol Genet.

[46] Nilsson E, Matte A, Perfilyev A, de Mello VD, Käkelä P, Pihlajamäki J (2015). Epigenetic alterations in human liver from subjects with type 2 diabetes in parallel with reduced folate levels. J Clin Endocrinol Metab.

[47] Kirchner H, Sinha I, Gao H, Kirchner H, Sinha I, Gao H (2016). Altered DNA methylation of glycolytic and lipogenic genes in liver from obese and type 2 diabetic patients. Mol Metab.

[48] Nicoletti CF, Nonino CB, de Oliveira BA, Pinhel MA, Mansego ML, Milagro MI (2016). DNA methylation and hydroxymethylation levels in relation to two weight loss strategies: energy-restricted diet or bariatric surgery. Obes Surg.

[49] Kirchner H, Nylen C, Laber S, Barrès R, Yan J, Krook A (2014). Altered promoter methylation of PDK4, IL1 B, IL6, and TNF after Roux-en Y gastric bypass. Surg Obes Relat Dis.

[50] Gloy VL, Briel M, Bhatt DL, Kashyap SR, Schauer PR, Mingrone G (2013). Bariatric surgery versus non-surgical treatment for obesity: a systematic review and meta-analysis of randomised controlled trials. BMJ.

[51] Barrès R, Osler ME, Yan J, Rune A, Fritz T, Caidahl K (2009). Non-CpG methylation of the PGC-1alpha promoter through DNMT3B controls mitochondrial density. Cell Metab.

[52] Nilsson EK, Ernst B, Voisin S, Almén MS, Benedict C, Mwinyi J (2015). Roux-en-Y gastric bypass surgery induces genome-wide promoter-specific changes in DNA methylation in whole blood of obese patients. PLoS ONE.

[53] Berglind D, Müller P, Willmer M, Sinha I, Tynelius P, Naslund E (2016). Differential methylation in inflammation and type 2 diabetes genes in siblings born before and after maternal bariatric surgery. Obesity.

[54] Ahrens M, Ammerpohl O, von Schönfels W, Kolarova J, Bens S, Itzel T (2013). DNA methylation analysis in nonalcoholic fatty liver disease suggests distinct disease-specific and remodeling signatures after bariatric surgery. Cell Metab.

[55] Benton MC, Johnstone A, Eccles D, Harmon B, Hayes MT, Lea RA (2015). An analysis of DNA methylation in human adipose tissue reveals differential modification of obesity genes before and after gastric bypass and weight loss. Genome Biol.

[56] Dahlman I, Sinha I, Gao H, Brodin D, Thorell A, Rydén M (2015). The fat cell epigenetic signature in post-obese women is characterized by global hypomethylation and differential DNA methylation of adipogenesis genes. Int J Obes.

[57] Lofgren P, Andersson I, Adolfsson B, Leijonhufvud BM, Hertel K, Hoffstedt J (2005). Long-term prospective and controlled studies demonstrate adipose tissue hypercellularity and relative leptin deficiency in the postobese state. J Clin Endocrinol Metab.

[58] Sethi JK, Vidal-Puig AJ (2007). Thematic review series: adipocyte biology. Adipose tissue function and plasticity orchestrate nutritional adaptation. J Lipid Res.

[59] Boström AE, Mwinyi J, Voisin S, Wu W, Schultes B, Zhang K (2016). Longitudinal genome-wide methylation study of Roux-en-Y gastric by-pass patients reveals novel CpG sites associated with essential hypertension. BMC Med Genom.

[60] Festa A, Williams K, Tracy RP, Wagenknecht LE, Haffner SM (2006). Progression of plasminogen activator inhibitor-1 and fibrinogen levels in relation to incident type 2 diabetes. Circulation.

[61] Ling C, Del Guerra S, Lupi R, Ro¨nn T, Granhall C, Luthman H (2008). Epigenetic regulation of PPARGC1A in human type 2 diabetic islets and effect on insulin secretion. Diabetologia.

[62] Yang BT, Dayeh TA, Kirkpatrick CL, Taneera J, Kumar R, Groop L (2011). Insulin promoter DNA methylation correlates negatively with insulin gene expression and positively with HbA(1c) levels in human pancreatic islets. Diabetologia.

[63] Yang BT, Dayeh TA, Volkov PA, Kirkpatrick CL, Malmgren S, Jing X (2012). Increased DNA methylation and decreased expression of PDX-1 in pancreatic islets from patients with type 2 diabetes. Mol Endocrinol.

[64] Liu ZH, Chen LL, Deng XL, Song HJ, Liao YF, Zeng TS (2012). Methylation status of CpG sites in the MCP-1 promoter is correlated to serum MCP-1 in type 2 diabetes. J Endocrinol Invest.

[65] Sala PC, Torrinhas RS, Heymsfield SB, Waitzberg DL (2012). Type 2 diabetes mellitus: a possible surgically reversible intestinal dysfunction. Obes Surg.

[66] Sala PC, Torrinhas RS, Giannella-Neto D, Waitzberg DL (2014). Relationship between gut hormones and glucose homeostasis after bariatric surgery. Diabetol Metab Syndr.

[67] Sala P, Belarmino G, Machado NM, Cardinelli CS, Al Assal K, Silva MM (2016). The SURMetaGIT study: design and rationale for a prospective pan-omics examination of the gastrointestinal response to Roux-en-Y gastric bypass surgery. J Int Med Res.

